# In vitro antidermatophytic activity of bioactive compounds from selected medicinal plants

**DOI:** 10.1186/s40543-021-00304-3

**Published:** 2021-11-03

**Authors:** Daisy Savarirajan, V. M. Ramesh, Arunachalam Muthaiyan

**Affiliations:** 1 Centre for Advanced Studies in Botany, University of Madras, Chennai, 600025 India; 2 Division of Arts and Sciences, University of New Mexico, Gallup, NM 87301 USA; 3 College of Science, Engineering and Technology, Grand Canyon University, 3300 W. Camelback Rd, Phoenix, AZ 85017 USA

**Keywords:** Antifungal activity, Dermatophytic fungi, Plant extracts, Dermatophytosis, *C*assia *occidentalis*, *Asparagus racemosus*, Anthraquinone, Saponin

## Abstract

Fungal infections are among the most difficult diseases to manage in humans. Eukaryotic fungal pathogens share many similarities with their host cells, which impairs the development of antifungal compounds. Therefore, it is desirable to harness the pharmaceutical potential of medicinal plants for antifungal drug discovery. In this study, the antifungal activity of sixteen plant extracts was investigated against selected dermatophytic fungi. Of the sixteen plants, the cladode (leaf) of *Asparagus racemosus,* and seed extract of *Cassia occidentalis* showed antifungal activity against *Microsporum gypseum*, *Microsporum nanum*, *Trichophyton mentagrophytes* and *Trichophyton terrestre*. The plant antifungal compounds were located by direct bioassay against *Cladosporium herbarum.* IR and NMR spectrometry analyses of these compounds identified the presence of saponin (in *A. racemosus*) and hydroxy anthraquinone (in *C. occidentalis*) in these antifungal compounds. The antidermatophytic activity of plant anthraquinone and saponins with reports of little or no hemolytic activity, makes these compounds ideal for alternative antifungal therapy and warrants further in-depth investigation in vivo.

## Introduction

Fungal infections are among the most difficult diseases to manage in humans. Reports indicate high rates of morbidity and mortality caused by fungal infections (CDC 2020). Despite their irreversible impact on human health, fungal pathogens have been mostly neglected by both the public and public health officials (Rodrigues and Nosanchuk 2020).

Dermatophytoses are characterized by superficial invasion by fungal hyphae in the skin, hair, and nails causing subacute or chronic infections (Burstein et al. 2020). Although dermatophyte infections are restricted to areas of the epidermis, they can be invasive and cause serious widespread infections in immunocompromised patients (Trottier et al. 2020). Major risk factors for the development of invasive fungal infections include, among others, HIV treatment in AIDS patients, cytotoxic chemotherapy in cancer patients, immunosuppressive therapy where innate defenses have been breached and the presence of catheters and other indwelling devices (Casadevall 2019; Li et al. 2020). Currently, mucormycosis also known as black fungus diseases, have been acquired as secondary infections in COVID-19 patients (Mahalaxmi et al. 2021).

As eukaryotic pathogens, fungi share many similarities with their host cells, which impairs the development of antifungal compounds (Rodrigues and Nosanchuk 2020). Cutaneous fungal parasites have survived several generations of therapeutic regimens, and the increasing invasive fungal infections along with the emerging resistance of pathogens and disadvantages with the existing antifungal drugs, demand the development of new antifungal drugs in clinical practice (Kim et al. 2020).

The antifungal potential of medicinal plants and their secondary metabolites against different fungal pathogens have been extensively studied (Chahal et al. 2021; Salhi et al. 2017; Mardani et al. 2018; Ezeonu et al. 2019; An et al. 2019; Simonetti et al. 2020; Alotibi et al. 2020; Kaur et al. 2021; Zagórska-Dziok et al. 2021). However, the limited armamentarium of the current classes of antifungal agents available (pyrimidine analogs, polyenes, azoles, and echinocandins), their toxicity, efficacy and the emergence of resistance are major bottlenecks limiting successful patient outcomes (Juvvadi et al. 2017; Chang et al. 2019). Plants contain a spectrum of secondary metabolites, such as phenols, coumarins, flavonoids, quinones, tannins and their glycosides, alkaloids, and essential oils. According to World Health Organization, medicinal plants would be the best source of a variety of drugs and numerous studies have been conducted on various medicinal plant extracts with the hope of discovering new and more efficient antifungal compounds (Scorzoni et al. 2017).

Plants have been widely used for their medicinal properties since ancient times (Nagy et al. 2014). Plants produce a lot of antioxidants and represent a source of novel compounds with promising antioxidant activity (Salanţă et al. 2014). *Acalypha indica* has been used for many therapeutics purposes such as anti-bacterial and other applications (Zahidin et al. 2017). *Achyranthus aspera* is used in treatment of cough, bronchitis and rheumatism, malarial fever, dysentery, asthma, hypertension and diabetes in traditional Indian medicine (Bhosale et al. 2012). *Annona reticulata* with anti-inflammatory effects is known to contain phytochemicals like tannins, alkaloids, phenols, glycosides, flavonoids and steroids (Jamkhande and Wattamwar 2015). *Annona squamosa leaves* have been studied for their biological activities, including anticancer, antidiabetic, antioxidant, and antimicrobial functions (DeFilipps and Krupnick 2018). *Asparagus racemosus* is a highly valued medicinal plant in Ayurvedic medicine system for the treatment of various ailments such as gastric ulcers, dyspepsia and cardiovascular diseases (Srivastava et al. 2018). *Cassia alata* is traditionally used in the treatment of ringworms, tinea infections, scabies, blotch, herpes, and eczema (Oladeji et al. 2020). Research findings indicate the therapeutic use of *Cassia fistula* as the rich source of antioxidant (Rahmani 2015). The antibacterial activities of leaves and rootbark extracts of *Cassia occidentalis* have been reported by Ibrahim et al. 2010. Upadhyay et al. 2014 reported the wound healing property of *Cleome viscosa*. *Feronia elephantum* fruit is traditionally used in India for the treatment for many liver disorders like jaundice (Sharma et al. 2012a, b). *Ficus religiosa* is used traditionally as antibacterial in the treatment of gonorrhea and skin diseases (Chandrasekar et al. 2010). *Lantana camara* is reported to have shown a wide range of antimicrobial activity (Kirimuhuzya et al. 2009). *Terminalia catapa* is often used in traditional medicine for the treatment of various infectious diseases (Ngouana et al. 2015). The wound healing potential of *Wedelia trilobata* leaves has been reported by Balekar et al. 2012. *Ziziphus jujuba* is known all around the world due to their health benefits, as both food and herbal medicine (Chen et al. 2017).

Therefore, in this study, the aforementioned sixteen plant specimens were selected and assayed for their antifungal activity against dermatophytic fungi in order to address the need for new antifungal agents with novel mode of action to be included as arsenals against drug resistant fungi.

## Methods

### Plant materials

A total of sixteen angiosperm plants, namely *Acalypa indica* (Euphorbiaceae), *Achyranthus aspera* (Amaranthaceae*), Annona reticulata* (Annonaceae)*, Annona squamosa* (Annonaceae), *Asparagus racemosus* (Asparagaceae*), Cassia alata* (Fabaceae*), Cassia fistula* (Fabaceae)*, Cassia occidentalis* (Fabaceae)*, Cleome viscosa* (Cleomaceae), *Feronia elephantum* (Rutaceae), *Ficus religiosa* (Moraceae)*, Lantana camera* (Verbenaceae), *Terminalia catapa* (Combretaceae), *Wedelia trilobata* (Asteraceae), and *Ziziphus jujuba* (Rhamnaceae), were collected from the university campus and around the city of Madras, Tamil Nadu, India. The plant samples were authenticated by Taxonomists from the Center for Advanced Studies in Botany, University of Madras, Tamil Nadu, India.

### Fungal Isolates

Test dermatophytic fungi *Micorsporum gypseum* MPS 199,901*, M. nanum* MPS 199,902*, Trichophyton mentagrophytes* MPS 199903 and *T. terrestre* MPS199904. (Ramesh and Hilda 1999; Anbu et al. 2004) were obtained from the Center for Advanced Studies in Botany, University of Madras, Chennai, India. These fungal specimens isolated from the city of Madras Parks and School playgrounds, were preserved in sterile distilled water at room temperature or in a cooling cabinet at 4℃ in the dark as described by Qiangqiang et al. (1998), Deshmukh (2002) and Nakasone et al (2004). The cultures were grown at 30 °C for 7 days on Potato Dextrose Agar and Sabouraud dextrose agar (SDA, HiMedia, India), respectively, prior to the antidermatophytic assay.

### Preparation of plant extracts

Fresh plant parts were washed, shade dried and then ground into powder using electric grinder. One hundred gram of powdered samples was extracted with 80% ethanol in a Soxhlet extractor for 72 h (Kalaivanan et al. 2013). The extracts were then passed through two layers of cheese cloth and then centrifuged at 3000 g for 10 min at 4 °C. The clear supernatant was collected and concentrated (to completely remove the solvent) in a rota-evaporator at 40 °C. The crude extracts were stored in a freezer at − 20 °C for further use.

### Antifungal activity of plant extracts

The antifungal effect of crude extract of test plants on the growth of dermatophytes was studied using Poisoned Food Technique (Jayshree et al. 2012). The crude extracts were dissolved in 10% aqueous dimethyl sulfoxide (DMSO), sterilized by filtration through a 0.45-μm membrane filter and assayed for their antifungal activity under aseptic condition. One milliliter of the test plant extract (in 10% DMSO) was mixed with 20 ml of sterilized Sabouraud dextrose agar (SDA) medium to yield a final concentration of 1 mg/ml. This mixture was immediately poured into Petri plates and allowed to solidify. The plates were inoculated in the center with 5-mm mycelia discs of 10-day-old test fungal culture. SDA medium was supplemented with reference antibiotic griseofulvin (MIC was 0.6 µg/ml for *M. gypseum*; 0.8 µg/ml for *M. nanum*; 2.5 µg/ml for *T. mentagrophytes*; and 0.6 µg/ml for *T. terrestre*) as positive control, while SDA medium with 10% DMSO devoid of plant extract served as negative control. The plates were incubated at 28 ± 2° C for 14 − 21 days. After incubation, the diameter of the fungal growth in control and sample plates was recorded and the percentage of mycelial inhibition (I %) was calculated as I % = [(dc − dt)/dc] X 100 (dc = colony diameter in control, dt = colony diameter in treatment) (Jayshree et al. 2012). All experiments were carried out in triplicates. The plant extracts showing high inhibitory effect were selected for further study.

### Phytochemical analysis of selected plant extracts

The crude extracts from cladodes of *A. racemosus* and seed of *C. occidentalis* were analyzed for the presence of alkaloids, phenolic compounds, tannins, cardiac glycosides, quinones, anthraquinones and saponins using standard methods listed as below (María et al. 2018; Trease and Evans 2002; Harbones 1998).

### Test for alkaloids

For alkaloids, 10 mg of each extract was treated with a few drops of Dragendorff’s reagent and Mayer’s reagent separately. The presence of alkaloids will be indicated by the formation of red–orange precipitate (Dragendorff) and yellowish-white precipitate.

### Test for phenols

To test the presence of phenolic compounds, 2 ml of alcoholic extract was treated with 5% ferric chloride. The presence of phenols will be indicated by the formation of a blue or black color.

### Test for tannins

The test for tannins was carried out with 0.1% FeCl3. Formation of brownish green or a blue-black color shows the presence of tannins.

### Test for saponins

The presence of saponins was tested by adding 0.5 g of crude extract to 2 ml of boiling water in a test tube and allowed to cool. The mixture was thoroughly shaken and observed for stable froth and further mixed with 3 drops of olive oil and shaken to observe formation of emulsion.

### Test for quinones

Borntrager’s test. The crude sample (20 mg) was treated with 3 mL of chloroform and the chloroform layer was separated. Then, 5% potassium hydroxide solution was added to the separated chloroform.

### Test for anthraquinones.

Modified Borntrager’s test. The crude extract (20 mg) was boiled with 3 ml of 10% hydrochloric acid for 3 min. The hot solution was filtered in a test tube, cooled and extracted gently with 3 ml of benzene. The upper benzene layer was pipetted off and shaken gently in a test tube with half of its volume of 10% ammonium hydroxide solution.

### Detection of antifungal compounds by direct bioassay

Plants that showed high antifungal activity in the initial screening were (100 g of powdered samples of the cladodes of *A. racemosus* and seeds of *C. occidentalis*) extracted with petroleum ether and ethanol in a Soxhlet extractor for 72 h (Kalaivanan et al. 2013). Then, the extracts were filtered, pooled and solvents were evaporated in rota-evaporator under reduced pressure at 45 °C, and the crude extracts were kept at 4 °C in refrigerator until further use. To detect the antifungal compounds, 100 μl of crude extracts (petroleum ether and ethanol) of *A. racemosus* and *C. occidentalis* were applied on a pre-activated (at 100 °C for 30 min) silica gel thin-layer chromatography (TLC) plates (Eastman 6069). The chromatogram was developed in a mixture of benzene and ethyl acetate (7:3) to a distance of 11 cm and dried at room temperature.

Antifungal compounds separated on TLC plates were bio-assayed according to the method described by Tabanca et al. (2005) with slight modification. Conidia from a week old *Cladosporium herbarum* growing on potato dextrose agar were harvested and washed by centrifugation. A highly concentrated conidial suspension was sprayed on thin chromatograms which were then oversprayed with half strength potato dextrose agar medium (PDA) at 45 °C. The sprayed chromatograms were incubated in moist chamber at room temperature for 48 h. The chromatograms were briefly air dried and sprayed with half strength PDA medium and incubated further in a moist chamber for 24 h at room temperature. At the end of the period, inhibitory area appeared as white zones on a green background of the mycelium. The Rf values of the antifungal zones were measured. Rf = distance traveled by sample/distance traveled by solvent.

### Antifungal assay of bioactive compounds

The antifungal compound detected by direct bioassay was eluted in respective solvents and loaded onto 9-mm filter paper discs (100 μg/disc). Petri plates containing Sabouraud dextrose agar medium were seeded with 0.1 ml of standardized spore suspension of test fungi, and the assay discs were aseptically transferred to Petri plates and incubated at room temperature (28 ± 1 °C) for a period of 7 days. The antifungal activity was assessed by measuring the zone of inhibition around the disc and compared with ketoconazole (15 μg/disc) control.

### Minimum inhibitory concentration assay

The minimum inhibitory concentrations (MICs) of antifungal compounds against dermatophytes were determined using the standard broth microdilution assay according to the guidelines of Clinical and Laboratory Standards Institute M38 (CLSI 2017). The test fungi were grown in Sabouraud Dextrose Agar (SDA) at 30 °C for 48 h and adjusted to a final density of 1 × 10^8^ CFU/mL by suspending in sterilized normal saline solution. The MIC values of test samples were determined as described by Kalaivanan et al. (2013) and Chellappandian et al. (2018). Twenty microliters of plant extract (50 mg/mL) in 10% DMSO was added to 980 µL of RPMI-1640 to yield the concentration 1000 µL (1 mg/mL). This solution was used for twofold serial dilutions to attain concentrations ranging from 3.9 to 1000 µg/mL. Two hundred microliters of the solution was placed into the first well of a 96-well microtiter plate, and then, 100 µl from the first well was transferred to the next well containing 100 µl of RPMI-1640. The same procedure was performed for all wells. A volume of 100 µL of standardized inoculum suspensions was transferred on to each well. Ketoconazole was used as positive antifungal control, and 10% DMSO was used as negative control. Each experiment was performed in triplicate and repeated twice. The MIC was interpreted as the lowest concentration of the test samples showing no visible growth.

### Spectrometric analysis of antifungal compounds

IR and NMR spectrum of the purified antifungal compounds from *A. racemosus* (cladodes) and *C. occidentalis* (seed) was analyzed using IR-P-983 spectrometer and EM-390 (90 MHz) NMR spectrometer as described by Ganesan and Mathuram (2020), and Akhtar et al. (2017). Deuterochloroform (CDCl_3_) was used as an internal reference for NMR studies.

### Statistical analysis

The results were expressed as the mean ± standard deviation. All statistical analyses were performed using SPSS version 27.0 statistical software, and comparison of treatments was carried out using one-way analysis of variance (ANOVA) and Turkey HSD test. P value < 0.05 was considered statistically significant.

## Results

### Antifungal activity of plant extracts against dermatophytes

**Fig. 1:**
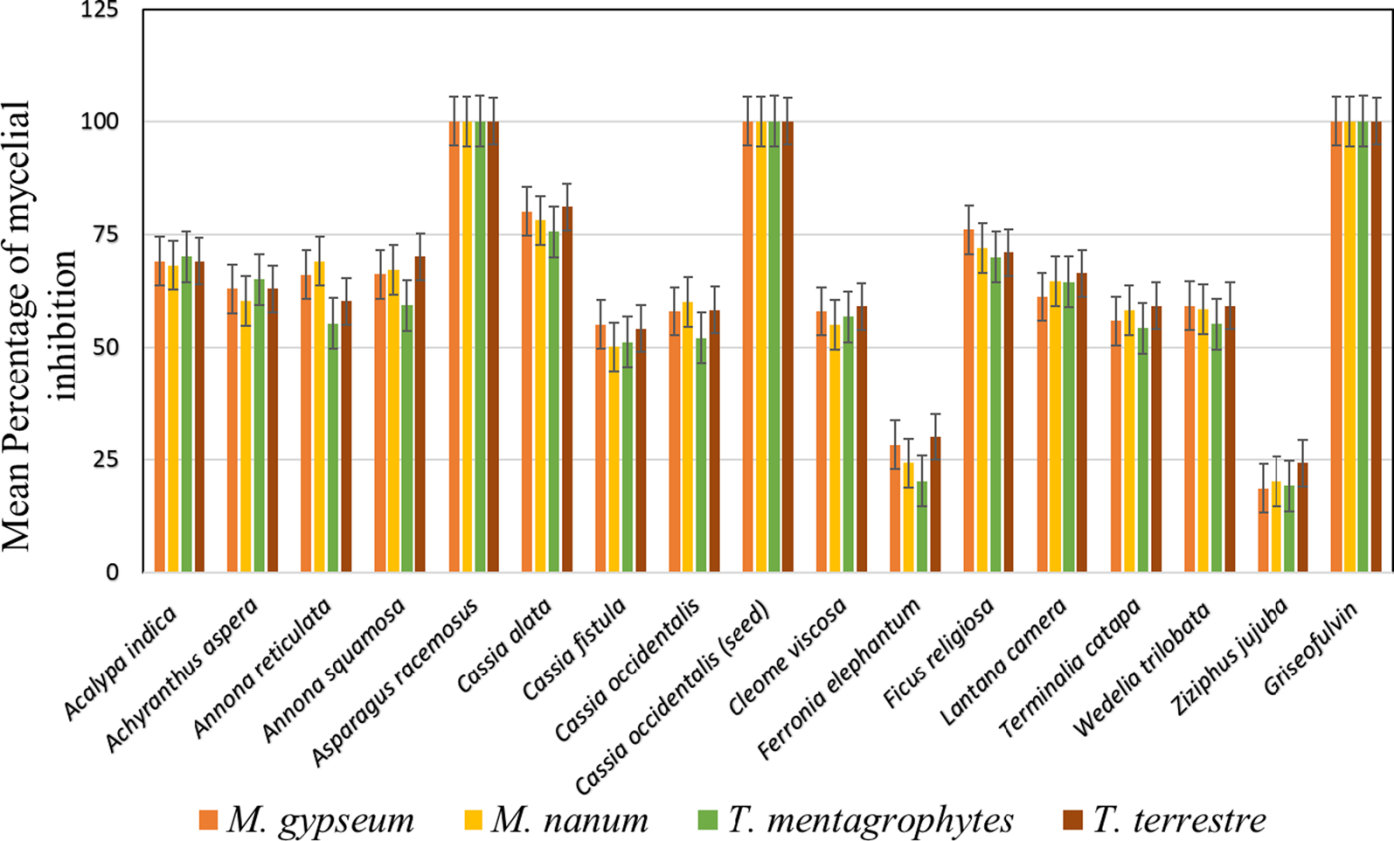
Antifungal evaluation of various plant extracts against dermatophytes. *Mean of three replica plates; ± standard deviation. **Significant at P < 0.05. ***Concentration of reference antibiotic (MIC) was 0.6 µg ml^−1^ for *M. gypseum*; 0.8 µg ml^−1^ for *M. nanum*; 2.5 µg ml^−1^ for *T. mentagrophytes*; and 0.6 µg ml^−1^ for *T. terrestre*

Of the sixteen plant species tested, crude ethanol extracts from cladodes (leaves) of *A. racemosus* and seed extract of *C. occidentalis* completely inhibited the mycelial growth of all four species of dermatophytes tested (Fig. 1). Compared to griseofulvin 0.6 µg/ml for *M. gypseum*; 0.8 µg/ml for *M. nanum*; 2.5 µg/ml for *T. mentagrophytes*; and 0.6 µg/ml for *T. terrestre* (positive controls), the leaf extracts of *A. indica, A. aspera, A. reticulata, A. squamosa, C. alata, F. religiosa* and *L. camera* exhibited about 60% inhibition, while the remaining plants showed low inhibitory effect against the test dermatophytes. The negative control (10% DMSO) showed no antifungal activity. Therefore, *A. racemosus* and *C. occidentalis* were selected for further study.

### Phytochemical analysis of plant extracts

Phytochemical screening of ethanolic extracts from the seed of *C. occidentalis* and the cladode of *A. racemosus* was carried out using various chemical assays in order to identify either the presence or absence of secondary metabolites that are useful in treating various ailments. Table 1 summarizes the phytochemicals present in assayed alcoholic extracts*.* Both extracts tested positive to Dragendorff (observing an orange precipitate) and Mayer’s test (formation of yellowish-white precipitate) indicating the presence of alkaloids. The seed extract of *C. occidentalis* also showed positive results for phenols, tannins, quinones and anthraquinones but was negative for saponins. The cladode of extract of *A. racemosus* tested positive for the presence of saponins, phenols and steroids but showed negative results for tannins, quinones and anthraquinones.

**Table 1:** Phytochemical analysis of alcoholic extracts from the cladode of *A. racemosus* and the seed *of C. occidentalis*. +  = present;− = absent

Phytochemicals	Plant extracts (ethanol)	
	Cladode of *A. racemosus*	Seed of *C. ocidentalis*
Steroids	** + **	** + **
Phenols	** + **	** + **
Tannins	**–**	** + **
Quinones	**–**	** + **
Anthraquinones	**–**	** + **
Saponins	** + **	**–**

### Detection of antifungal compounds by direct bioassay

**Fig. 2:**
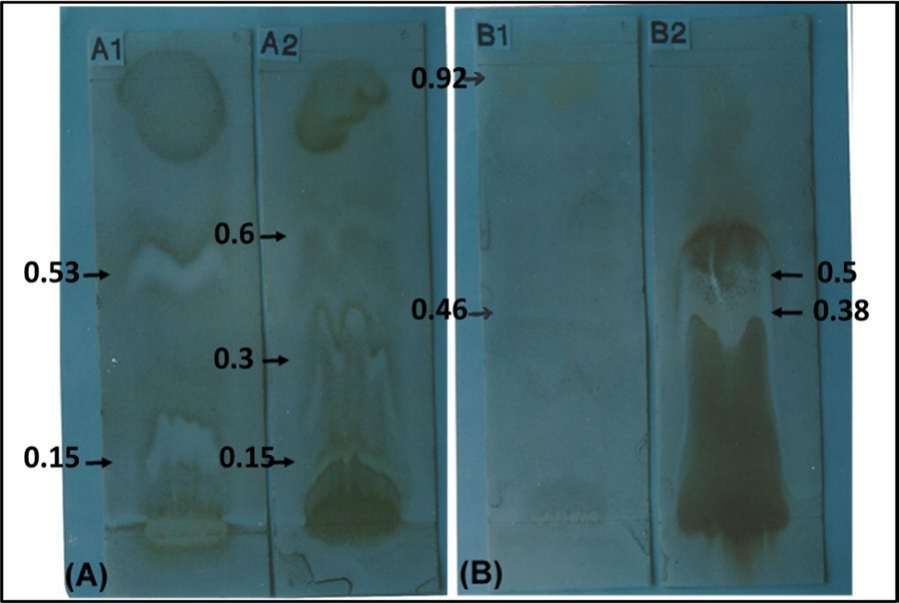
Detection of antifungal compounds in cladode extract of *A. racemosus*. (**a**) and in seed extract of *C. occidentalis* (**b**) by direct bioassay. A1—petroleum ether extract; A2—ethanol extract; B1—petroleum ether extract; B2—ethanol extract

The Rf values of the antifungal zones on the TLC plates were measured (Fig. 2 Plates A and B)**.** For *A. racemosus*, the petroleum ether extract (A1) showed two antifungal zones at Rf values 0.53 and 0.15, whereas ethanol extract (A2) showed three antifungal zones at Rf values of 0.6, 0.3, 0.15. In the case of *C. occidentalis*, the petroleum ether extract (B1) showed two antifungal zones at Rf values 0.92 and 0.46 and ethanol extract (B2) also exhibited two antifungal zones at Rf values 0.38 and 0.3 against test fungi (*Cladosporium herbarum)*.

The antifungal effect of TLC detected compounds against dermatophytic fungi is presented in Table 2. The antifungal compounds in petroleum ether extract from *A. racemosus* showed no inhibitory effect against any of the test dermatophytes, whereas the antifungal compounds from ethanol extract detected at the Rf value of 0.3 were inhibitory to all the test fungi**.** The antifungal compounds detected at the Rf value of 0.92 from petroleum ether extract of *C. occidentalis* showed inhibitory effect against all the dermatophytes tested, whereas compounds at Rf 0.46 showed no inhibitory effect against any of the test dermatophytes (Table 2)**.** The antifungal compounds from ethanol extract also showed no inhibitory effect against any the test dermatophytes. Therefore, the antifungal compounds showing inhibitory effect from *C. occidentalis* (Rf 0.92) and *A. racemosus* (Rf 0.3) were chosen for further characterization.

**Table 2:** Efficacy of antifungal zones located on TLC

Fungal species	The antifungal effect of TLC detected compounds at different Rf values								
	*A. racemosus*					*C. occidentalis*			
	Petroleum ether extract		Ethanol extract			Petroleum ether extract		Ethanol extract	
	0.53	0.15	0.6	0.3	0.15	0.92	0.46	0.38	0.3
*M. gypseum*	–	–	–	+	–	+	–	–	–
*M. nanum*	–	–	–	+	–	+	–	–	–
*T. mentagrophytes*	–	–	–	+	–	+	–	–	–
*T. terrestre*	–	–	–	+	–	+	–	–	–

Rf, Retention factor; +  = exhibits inhibitory effect;− = exhibits no inhibitory effect

### Minimum inhibitory concentration assay

Evaluation of MIC of TLC detected antifungal compounds *C. occidentalis* and *A. racemosus* indicated variable inhibitory effects (Table 3)**.** The MICs of the two bioactive compounds were ranging from 15.62 to 62.5 μg/mL. Among all the tested fungi, *M. gypseum* was highly sensitive to both compounds, followed by *M. canis* and *T. terestre*.

**Table 3:** Minimum inhibitory concentration of TLC detected bioactive compounds from *A. racemosus* and *C. occidentalis* against dermatophytes

Dermatophytes	Minimum inhibitory concentration (µg/mL) of TLC detected compounds	
	*A. racemosus* (Rf 0.3)	*C. Occidentalis* (Rf 0.92)
*M. gypseum*	31.25	15.62
*M. nanum*	62.5	31.25
*T. mentagrophytes*	62.5	62.5
*T. terestre*	31.25	31.25

### IR spectrum of antifungal compounds from cladode extract of *A. racemosus* and seed extract *C. occidentalis*

**Fig. 3:**
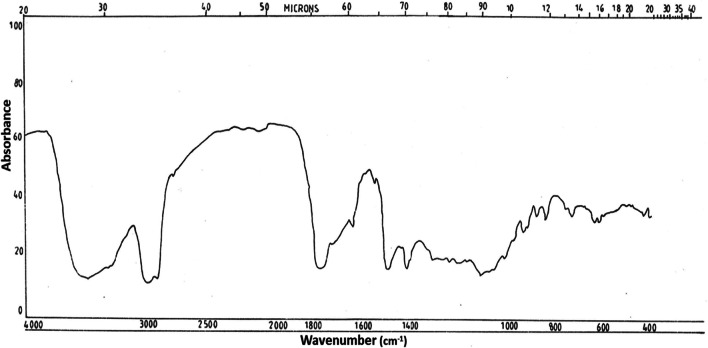
Infrared spectrum of antifungal compound (Saponin) from *A. racemosus*

The TLC detected antifungal compounds were partially characterized using IR and NMR spectral studies. The IR spectrum of antifungal compound from *A. racemosus* showed a broad peak at 3550–3450 cm^−1^ indicating the presence of OH group (Fig. 3). Formation of peaks around 920, 890 and 840 showed the presence of typical spirochetal side chain suggesting that it is spirostanol glycosides.

**Fig. 4:**
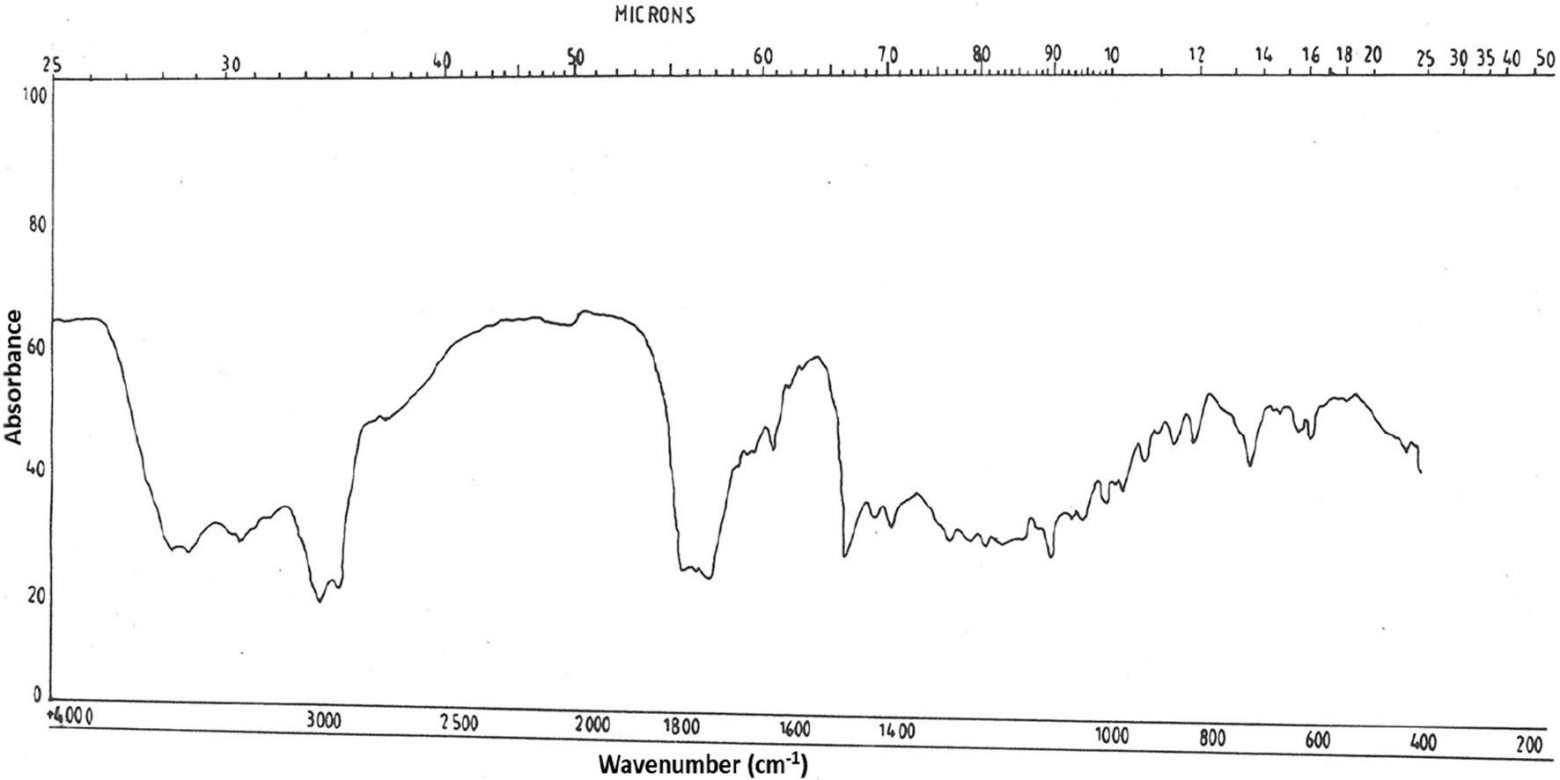
Infrared spectrum of antifungal compound (dihydroxyanthraquinone) from *C. occidentalis*

The IR spectrum of antifungal compound from *C. occidentalis* showed 2 broad peaks at 3480 cm^−1^ and 3580 cm^−1^ indicating the presence of phenolic OH (Fig. 4). Peaks at 2990 cm^−1^ showed the presence of C-H stretching frequency. The presence of aromatic skeleton was shown by the formation peaks at 3000, 1500, 820 and 740 cm^−1^. The absorption peak at 1640 cm^−1^ showed the presence of (carboxyl group) carbonyl band.

### NMR spectrum of antifungal compounds from cladode extract of *A. racemosus* and seed extract *C. occidentalis*

**Fig. 5:**
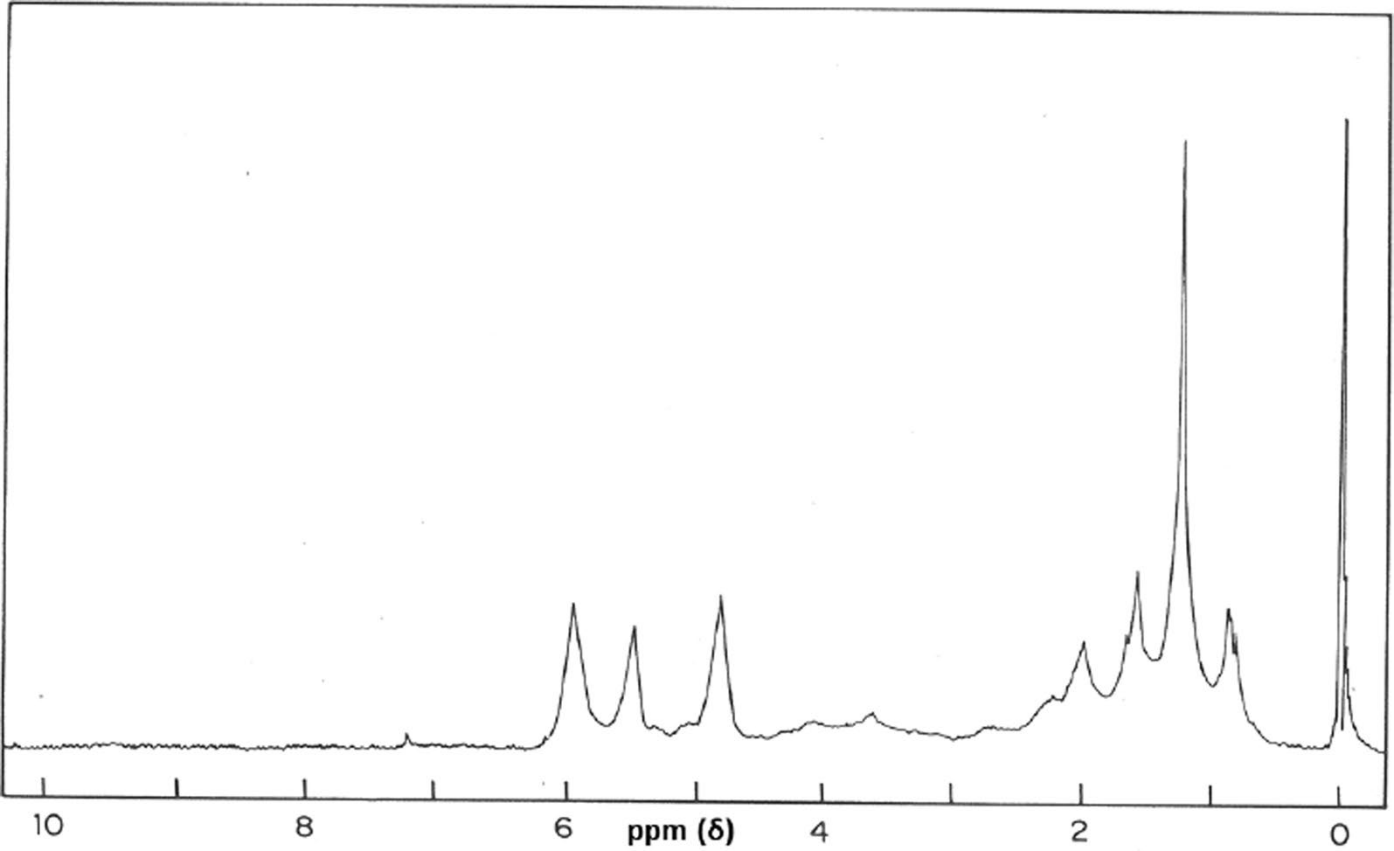
NMR spectrum of saponin from *A. racemosus*

**Fig. 6:**
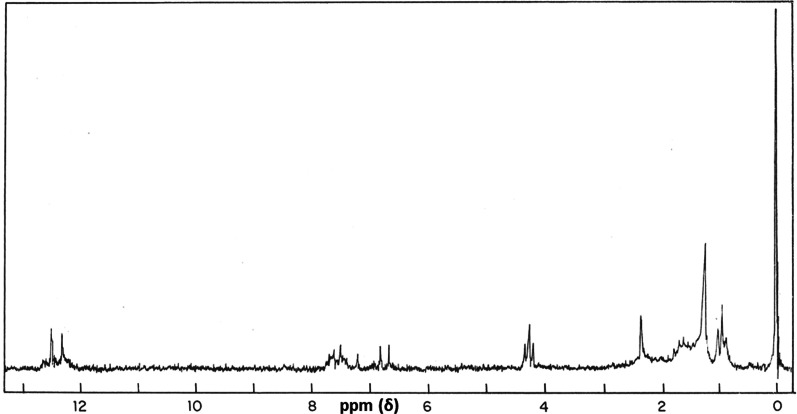
NMR spectrum of dihydroxyanthraquinone from *C. occidentalis*

The NMR spectrum of antifungal compound from *A. racemosus* showed signals at ó (ppm) 0.8, 0.90, 1.35, 1.65, 4.85, 5.43 and 5.88, which are characteristic of spirostanol saponin (Fig. 5). The NMR spectrum of compound from *C. occidentalis* (RF 0.92) showed signals at ó (ppm) 2.35, 4.30, 6.70, 6.88, 7.25 and signals at 12.3 and 12.5 which are typical of phenolic OH group (Fig. 6).

## Discussion

Fungal members belonging to various taxonomical groups detected in the soil of areas of various human activities are potentially pathogenic to human and animals (Wójcik 2016). Some of them are considered as emerging pathogens (Wójcik 2016; Friedman and Schwartz 2019; Morio 2020). Reports indicate health concerns due to public parks and school playgrounds as ideal environment for the growth of pathogenic fungal dermatophytes (Dehghan et al. 2019; Taghipour et al. 2021). Especially, prevalence of dermatophytic infections is more common in tropical countries due to high temperature, humidity and sweating (de Albuquerque Maranhão et al. 2019; Araya et al. 2021; Khodadadi et al. 2021). Besides the evolutionary pressure from the antifungal drugs, it is now identified that environmental pressures including the climate change are affecting the fungal evolution to confer novel traits including virulence and antifungal resistance (Nnadi and Carter 2021). Human-dominated environments like parks or playground are natural niches of dermatophytic fungi and pose a public health problem. Hence, in the search for alternative therapies for the emerging resistant fungal strains, we investigated the potential antifungal effect of several plant extracts on dermatophytes isolated from public parks and school playgrounds.

The different parts of *C. occidentalis* and *A. racemosus* such as root, leaves, seeds and pods have been used in traditional medicines for the treatment of various infectious and non-infectious diseases (Fidèle et al. 2017; Karuna et al. 2017; Srivastava et al. 2018; Okonkwo et al. 2019). In this present study, the extracts of *C. occidentalis* (seed) and *A. racemosus* (cladode) inhibited the growth of all the tested dermatophytic fungi (Table 1).

Similarly, the antifungal activity against fluconazole resistant *Candida* isolates was reported in *Cassia fistula* (Sony et al. 2018). *Cassia fistula* seed extract reportedly disrupts the cell membrane in *C. albicans*, resulting in damage to yeast cells (Sitarek et al. 2020a, b). Onlom et al. (2014) reported the in vitro antifungal activity of *A. racemosus* roots extracts against *Malassezia* spp. and its potential use as an active ingredient in an anti-dandruff formulation.

Medicinal plants produce a diverse range of bioactive molecules that provide reliable therapy in the treatment of various diseases and skin infections in humans (Sitarek et al. 2020a, b). The phytochemical analysis of the ethanolic extract of *C. occidentalis* indicated the presence of phenols, tannins, quinones and anthraquinones but was negative for saponins. The cladode extract of *A. racemosus*, tested positive for the presence of saponins but did not contain phenols, tannins, quinones and anthraquinones. Previous studies reported that the presence of saponins and flavonoids in these plants plays a role in therapeutic applications (Onlom et al. 2017; Srivastava et al. 2018; Issa et al. 2020).

The plant kingdom has an immense potential for the safe use of natural products in treatment of invasive and systemic fungal infections (D'agostino et al. 2019). Evaluation of MIC of the TLC detected bioactive compounds, *C. occidentalis* and *A. racemosus*, indicated variable inhibitory effects against fungal pathogens (Table 3). Among all the tested fungal specimens, *M. gypseum* was highly sensitive to both compounds from *C. occidentalis* and *A. racemosus*, followed by *M. nanum* and *T. terestre*. When compared to the antifungal activity of antibiotics reported by Banfalvi (2020), *M. gypseum* was more susceptible to gentamicin B1 (MIC 3.1 µg/mL). However, evidence shows antifungal topicals are more expensive when used in combination with corticosteroid and less effective as single-agent antifungals (Wheat et al. 2017). Therefore, the synergistic potential of the test plant extracts with topical antifungals that are already in medical use, makes it a promising alternative for antifungal therapy. This approach could help curb the rise in chronic and recalcitrant dermatophytosis cases (Tuknayat et al. 2020).

In the present study, fractions from *C. occidentalis* and *A. racemosus* that were inhibitory to the test dermatophytes were partially characterized by IR, NMR and identified as dihydroxy anthraquinone and spirostanol saponin, respectively. The compound from the cladodes of *A. racemosus* was identical in spectral properties (IR and NMR) with those reported for spirostanol saponin (Sharma et al. 2012a, b; Sharma et al. 2009). Therefore, it is concluded that the compound isolated from the ethanol extract of *A. racemosus* (cladode) may be spirostanol saponin. Saponins perforate lipid bilayers and increase the permeability of the cell membrane allowing transport of molecules that would otherwise be excluded (Chen et al. 2017; Efimova and Ostroumova 2021). The antidermatophytic activity of spirostanol saponin isolated from *A. racemosus* cladode in our study is similar to other findings. Antimycotic activity of spirostanol saponins has been reported in *Solanum hispidum* leaves (Manases González et al. 2004; Diretto et al. 2017; El Sayed et al. 2020; Dąbrowska-Balcerzak et al. 2021). Therefore, this plant saponin has great potential to bolster the antifungal armamentarium for treatment of fungal infections that does not respond to conventional therapy.

The IR and NMR spectrum data for the antifungal compound from the seeds of *C. occidentalis*, inferred that the compound has anthraquinone skeleton which was identical in spectral properties to those reported for dihydroxy methyl anthraquinone derivative (Tiwari and Singh 1979; Mehta 2012). According to Brilhante, (2020), the antifungal mechanism of anthraquinones may be related to the inhibition of (1,3)-β-D-glucan synthase activity, leading to disruption of (1,3)-β-D-glucans in the fungal cell wall. High antifungal activity of anthraquinone aglycones was reported against clinical strains of dermatophytes in *Senna alata* leaves, and *C. fistula* pod pulp (Wuthi-udomlert et al. 2010; Chewchinda et al. 2013; Friedman et al. 2020) reported the inhibitory potency of plant derived anthraquinones against pathogenic fungi.

The antidermatophytic activity of anthraquinone and saponins has been confirmed by several studies (Njateng et al. 2013; Yang et al. 2019). Phytochemical screening of *Polyscias fulva* indicated antidermatophytic activity of saponins, tannins, alkaloids, and anthraquinones (Njateng et al. 2013). Reports on *Cassia occidentalis* poisoning are rare (Chappola et al. 2018). Moreover, to avoid unwanted side effects, natural anthraquinones are preferred as an alternative antifungal. Hence, *Cassia occidentalis*, a traditional Ayurvedic edible shrub with anthraquinones as the principle active constituent, warrants further investigation for development of novel antifungals for treatment of filamentous fungal dermatophytes.

Climate changes can affect epidemiology of fungal disease, leading to the emergence of new virulent strains (van Rhijn and Bromley 2021). Conventional antifungal drugs like griseofulvin gets quickly eliminated from the body and must be taken over an extended period to have efficacy and therefore have adverse side effects (Gupta et al. 2018). Therefore, the quest for alternative therapies with new antimicrobial mechanisms has become an urgent priority to overcome the developing resistance of fungal pathogens (Shaban et al. 2020). Plants are an indispensable source of novel compounds that can be used for the treatment of multidrug-resistant (MDR) fungal infections (Marquez and Quave 2020). Needless to say, novel antifungal agents in the drug development pipeline hold a promising future for antifungal therapeutics (Wall and Lopez-Ribot 2020).

## Conclusions

In conclusion, the results demonstrated that extracts from *A. racemosus* cladodes and seed of *C. occidentalis* have strong antifungal activity against dermatophytic fungal species, *M. gypseum*, *M. nanum*, *T. mentagrophytes* and *T*. *terrestre.* Partial characterization of the antifungal compounds from these two plant species using IR and NMR inferred the two compounds from *C. occidentalis* as hydroxy anthraquinone and from *A. racemosus*, as saponin. Conventional antifungal agents have limited effectiveness due to their common side effects. The antidermatophytic activity of plant anthraquinone and saponins with reports of little or no hemolytic activity, makes these compounds ideally suited for alternative antifungal therapy. The precise determination of selective antifungal activity of hydroxy anthraquinone and saponin warrants future in-depth investigation in vivo. The results of the present investigation support the traditional use of the selected medicinal plants in the treatment of various infections. They also provide an important basis for further studies to test the clinical safety and efficacy of the phytotherapeutic bioactive compounds. Comparison of the antifungal effect on multiple fungal strains along with a standard fungal strain would be necessary to provide additional valid information for the future therapeutic applications against other fungal pathogens.

## Data Availability

All data generated or analyzed during this study are included in this published article.

## Abbreviations

SDA: Sabouraud dextrose agar
SDB: Sabouraud dextrose broth
DMSO: Dimethyl sulfoxide
CFU: Colony forming units
MIC: Minimum inhibitory concentration
PDA: Potato dextrose agar
TLC: Thin-layer chromatography
RF: Relative to front
CLSI: Clinical and Laboratory Standards Institute
RPMI: Roswell Park Memorial Institute 1640 Medium
IR: Infra-red spectroscopy
NMR: Nuclear magnetic resonance spectroscopy

## References

[CR1] Alotibi FO, Ashour EH, Al-Basher G (2020). Evaluation of the antifungal activity of *Rumex vesicarius* L. and *Ziziphus spina-christi* (L) Desf. Aqueous extracts and assessment of the morphological changes induced to certain myco-phytopathogens. Saudi J Biol Sci.

[CR2] An A, Je A, Cb U, Mn I (2019). Identification and control of specific aflatoxin-producing fungi in stored maize seeds in awka using *Azadirachta indica* (neem) and garcinia kola seeds. Pak J Pharm Sci.

[CR3] Anbu P, Hilda A, Gopinath SC (2004). Keratinophilic fungi of poultry farm and feather dumping soil in Tamil Nadu. India Mycopathologia.

[CR4] Ankad BS, Mukherjee SS, Nikam BP, Reshme AS, Sakhare PS, Mural PH (2020). Dermoscopic characterization of dermatophytosis: a preliminary observation. Indian Dermatol Online J.

[CR5] Araya S, Tesfaye B, Fente D (2020). Epidemiology of dermatophyte and non-dermatophyte fungi infection in Ethiopia. Clin Cosmet Investig Dermatol.

[CR6] Araya S, Abuye M, Negesso AE (2021). Epidemiological characterization of dermatomycosis in Ethiopia. Clin Cosmet Investig Dermatol.

[CR7] Balekar N, Katkam NG, Nakpheng T, Jehtae K, Srichana T (2012). Evaluation of the wound healing potential of *Wedelia trilobata* (L.) leaves. J Ethnopharmacol.

[CR8] Banfalvi G (2020). Antifungal activity of gentamicin B1 against systemic plant mycoses. Molecules (basel, Switzerland).

[CR9] Bhosale UA, Yegnanarayan R, Pophale P, Somani R (2012). Effect of aqueous extracts of *Achyranthes aspera* Linn. on experimental animal model for inflammation. Anc Sci Life.

[CR10] Biasi-Garbin RP, Demitto FO, do Amaral RCR, Ferreira MRA, Soares LAL, Svidzinski TIE, Yamada-Ogatta SF (2016). Antifungal potential of plant species from Brazilian caatinga against dermatophytes. Rev Inst Med Trop Sao Paulo.

[CR11] Brilhante RSN, Araújo GDS, Fonseca XMQC, Guedes GMM, Aguiar L, Castelo-Branco DSCM, Cordeiro RA, Sidrim JJC, Pereira Neto WA, Rocha MFG (2020). Antifungal effect of anthraquinones against *Cryptococcus neoformans*: detection of synergism with amphotericin B. Med Mycol.

[CR12] Buil JB, Meijer E, Denning DW, Verweij PE, Meis JF (2020). Burden of serious fungal infections in the Netherlands. Mycoses.

[CR13] Burstein VL, Beccacece I, Guasconi L, Mena CJ, Cervi L, Chiapello LS (2020). Skin immunity to dermatophytes: from experimental infection models to human disease. Front Immunol.

[CR14] Casadevall A (2019). Global catastrophic threats from the fungal kingdom: fungal catastrophic threats. Curr Top Microbiol Immunol.

[CR100] CDC. Burden of fungal diseases in the United States. Fungal diseases. 2020. https://www.cdc.gov/fungal/cdc-and-fungal/burden.html. Accessed 8 Dec 2020

[CR15] Chahal R, Nanda A, Akkol EK, Sobarzo-Sánchez E, Arya A, Kaushik D, Dutt R, Bhardwaj R, Rahman MH, Mittal V (2021). *Ageratum conyzoides* L. and its secondary metabolites in the management of different fungal pathogens. Molecules.

[CR16] Chandrasekar SB, Bhanumathy M, Pawar AT, Somasundaram T (2010). Phytopharmacology of *Ficus religiosa*. Pharmacogn Rev.

[CR17] Chang Z, Yadav V, Lee SC, Heitman J (2019). Epigenetic mechanisms of drug resistance in fungi. Fungal Genet Biol.

[CR18] Chen J, Liu X, Li Z, Qi A, Yao P, Zhou Z, Dong TTX, Tsim KWK (2017). A review of dietary *Ziziphus jujuba* fruit (jujube): developing health food supplements for brain protection. Evid Based Compl Alternat Med.

[CR19] Chappola V, Kanwal SK, Sharma AG, Kumar V (2018). Hepatomyoencephalopathy secondary to cassia occidentalis poisoning: report of three cases from North India. Indian J Crit Care Med Peer-Revi.

[CR20] Chellappandian M, Saravanan M, Pandikumar P, Harikrishnan P, Thirugnanasambantham K, Subramanian S, Hairul-Islam VI, Ignacimuthu S (2018). Traditionally practiced medicinal plant extracts inhibit the ergosterol biosynthesis of clinically isolated dermatophytic pathogens. J Mycol Med.

[CR21] Chen M, Balhara V, Jaimes Castillo AM, Balsevich J, Johnston LJ (2017). Interaction of saponin 1688 with phase separated lipid bilayers. Biochim Biophys Acta Biomembr.

[CR22] Chewchinda S, Wuthi-udomlert M, Gritsanapan W (2013). HPLC quantitative analysis of Rhein and antidermatophytic activity of cassia fistula pod pulp extracts of various storage conditions. Biomed Res Int.

[CR23] Chikoi R, Nyawale HA, Mghanga FP (2018). Magnitude and associated risk factors of superficial skin fungal infection among primary school children in Southern Tanzania. Cureus.

[CR24] CLSI Reference Method for Broth Dilution Antifungal Susceptibility Testing of Filamentous Fungi (2017) 3rd ed. CLSI standard M38. Wayne, PA: Clinical and Laboratory Standards Institute.

[CR25] D'agostino M, Tesse N, Frippiat JP, Machouart M, Debourgogne A (2019). Essential oils and their natural active compounds presenting antifungal properties. Molecules (basel, Switzerland).

[CR26] de Albuquerque Maranhão FC, Oliveira-Júnior JB, Dos Santos Araújo MA, Silva D (2019). Mycoses in northeastern Brazil: epidemiology and prevalence of fungal species in 8 years of retrospective analysis in Alagoas. Braz J Microbiol.

[CR27] DeFilipps RA, Krupnick GA (2018). The medicinal plants of Myanmar. PhytoKeys.

[CR28] Dąbrowska-Balcerzak K, Nartowska J, Wawer I, Siudem P, Paradowska K (2021). Spirostanol sapogenins and saponins from *Convallaria majalis* L. structural characterization by 2D NMR, theoretical GIAO DFT calculations and molecular modeling. Molecules (basel, Switzerland).

[CR29] Dehghan P, Yousefi Jalali S, Chadeganipour M (2019). Frequency distribution of keratinophilic dermatophyte fungi from the soil of different zones in Isfahan using morphological and molecular methods. Adv Biomed Res.

[CR30] Deshmukh SK (2002). The maintenance and preservation of keratinophilic fungi and related dermatophytes. Mycoses.

[CR31] Diretto G, Rubio-Moraga A, Argandoña J, Castillo P, Gómez-Gómez L, Ahrazem O (2017). Tissue-specific accumulation of sulfur compounds and saponins in different parts of garlic cloves from purple and white ecotypes. Molecules (basel, Switzerland).

[CR32] Efimova SS, Ostroumova OS (2021). Is the membrane lipid matrix a key target for action of pharmacologically active plant saponins?. Int J Mol Sci.

[CR33] El Sayed AM, Basam SM, El-Naggar E, Marzouk HS, El-Hawary S (2020). LC-MS/MS and GC-MS profiling as well as the antimicrobial effect of leaves of selected Yucca species introduced to Egypt. Sci Rep.

[CR34] Ezeonu CS, Tatah VS, Imo C, Mamma E, Mayel MH, Kukoyi AJ, Jeji IA (2019). Inhibitory effect of aqueous and ethanolic extracts of neem parts on fungal rot disease of *Solanum tuberosum*. Pak J Biol Sci.

[CR35] Faustino C, Pinheiro L (2020). Lipid systems for the delivery of amphotericin B in antifungal therapy. Pharmaceutics.

[CR36] Fernández de Ullivarri M, Arbulu S, Garcia-Gutierrez E, Cotter PD (2020). Antifungal peptides as therapeutic agents. Front Cell Infect Microbiol.

[CR37] Fidèle N, Joseph B, Emmanuel T, Théophile D (2017). Hypolipidemic, antioxidant and anti-atherosclerogenic effect of aqueous extract leaves of *Cassia occidentalis* Linn. (Caesalpiniaceae) in diet-induced hypercholesterolemic rats. BMC Complement Altern Med.

[CR38] Friedman DZP, Schwartz IS (2019). Emerging fungal infections: new patients, new patterns, and new pathogens. J Fungi (basel).

[CR39] Friedman M, Xu A, Lee R, Nguyen DN, Phan TA, Hamada SM, Panchel R, Tam CC, Kim JH, Cheng LW, Land KM (2020). The inhibitory activity of anthraquinones against pathogenic protozoa, bacteria, and fungi and the relationship to structure. Molecules (basel, Switzerland).

[CR40] González M, Zamilpa A, Marquina S, Navarro V, Alvarez L (2004). Antimycotic spirostanol saponins from *Solanum hispidum* leaves and their structure−activity relationships. J Nat Prod.

[CR41] Gupta AK, Mays RR, Versteeg SG, Piraccini BM, Shear NH, Piguet V, Tosti A, Friedlander SF (2018). Tinea capitis in children: a systematic review of management. J Eur Acad Dermatol Venereol.

[CR120] Harbone JB (1998). Phytochemical methods—a guide to modern techniques of plant analysis.

[CR42] Ibrahim MA, Aliyu AB, Sallau AB, Bashir M, Yunusa I, Umar TS (2010). Senna occidentalis leaf extract possesses antitrypanosomal activity and ameliorates the trypanosome-induced anemia and organ damage. Pharmacognosy Res.

[CR43] Issa TO, Mohamed Ahmed AI, Mohamed YS, Yagi S, Makhawi AM, Khider TO (2020). Physiochemical, insecticidal, and antidiabetic activities of *Senna occidentalis* Linn root. Biochem Res Int.

[CR44] Jamkhande PG, Wattamwar AS (2015). *Annona reticulata* Linn. (Bullock's heart): plant profile, phytochemistry and pharmacological properties. J Tradit Complement Med.

[CR45] Jayshree D, Jha DK, Policegoudra RS, Afjal HM, Mrinmoy D, Chattopadhyay P, Singh L (2012). Isolation and characterization of antidermatophytic bioactive molecules from *Piper longum* L. Leaves Indian J Microbiol.

[CR46] Juvvadi PR, Lee SC, Heitman J, Steinbach WJ (2017). Calcineurin in fungal virulence and drug resistance: Prospects for harnessing targeted inhibition of calcineurin for an antifungal therapeutic approach. Virulence.

[CR47] Kalaivanan C, Chandrasekaran M, Venkatesalu V (2013). Screening of selected medicinal plants for in vitro antidermatophytic activity. J Mycol Med..

[CR48] Karuna DS, Dey P, Das S, Kundu A, Bhakta T (2017). In vitro antioxidant activities of root extract of *Asparagus racemosus* Linn. J Tradit Complement Med.

[CR49] Kaur N, Bains A, Kaushik R, Dhull SB, Melinda F, Chawla P (2021). A review on antifungal efficiency of plant extracts entrenched polysaccharide-based nanohydrogels. Nutrients.

[CR50] Khodadadi H, Zomorodian K, Nouraei H, Zareshahrabadi Z, Barzegar S, Zare MR, Hir K (2021). Prevalence of superficial-cutaneous fungal infections in Shiraz, Iran: a five-year retrospective study (2015–2019). J Clin Lab Anal.

[CR51] Kim JH, Cheng LW, Chan KL, Tam CC, Mahoney N, Friedman M, Shilman MM, Land KM (2020). Antifungal drug repurposing. Antibiotics (basel, Switzerland).

[CR52] Kirimuhuzya C, Waako P, Joloba M, Odyek O (2009). The anti-mycobacterial activity of *Lantana camara* a plant traditionally used to treat symptoms of tuberculosis in South-western Uganda. Afr Health Sci.

[CR53] Li X, Lau SK, Woo PC (2020). Fungal infection risks associated with the use of cytokine antagonists and immune checkpoint inhibitors. Exp Biol Med (maywood).

[CR54] Mahalaxmi I, Jayaramayya K, Venkatesan D, Subramaniam MD, Renu K, Vijayakumar P, Narayanasamy A, Gopalakrishnan AV, Kumar NS, Sivaprakash P, Sambasiva RK, Vellingiri B (2021). Mucormycosis: an opportunistic pathogen during COVID-19. Environ Res.

[CR55] Mardani M, Badiee P, Gharibnavaz M, Jassebi A, Jafarian H, Ghassemi F (2018). Comparison of anti-Candida activities of the ancient plants *Lawsonia inermis* and *Ziziphus spina christi* with antifungal drugs in Candida species isolated from oral cavity. J Conserv Dent.

[CR56] María R, Shirley M, Xavie C, Jaime S, David V, Rosa S, Jodie D (2018). Preliminary phytochemical screening, total phenolic content and antibacterial activity of thirteen native species from Guayas province Ecuador. J King Saud Univ Sci.

[CR57] Marquez L, Quave CL (2020). Prevalence and therapeutic challenges of fungal drug resistance: role for plants in drug discovery. Antibiotics (basel, Switzerland).

[CR58] Mehta JP (2012). Separation and characterization of Anthraquinone derivatives from Cassia fistula using chromatographic and spectral techniques. Int J Chem Sci.

[CR59] Morio F (2020). Dear medical mycologists, it is time to look outside the box. FEMS Yeast Res.

[CR60] Nagy M, Tofană M, Socaci S, Pop A, Borș M, Fărcaș A, Moldovan O (2014). Total phenolic, flavonoids and antioxidant capacity of some medicinal and aromatic plants. Bulletin of University of Agricultural Sciences and Veterinary Medicine Cluj-Napoca. Food Sci Technol.

[CR61] Nakasone KK, Peterson SW, Jong S-C, Mueller GM, Bills GF, Foster MS (2004). Preservation and distribution of fungal cultures. Biodiversity of fungi: inventory and monitoring methods.

[CR62] Nnadi NE, Carter DA (2021). Climate change and the emergence of fungal pathogens. PLoS Pathog.

[CR63] Ngouana TK, Mbouna CDJ, Kuipou RMT, Tchuenmogne MAT, Zeuko'o EM, Ngouana V, Mallié M, Bertout S, Boyom FF (2015). Potent and synergistic extract combinations from *Terminalia Catappa*, *Terminalia Mantaly* and *Monodora tenuifolia* against pathogenic yeasts. Medicines (basel).

[CR64] Njateng GSS, Gatsing D, Mouokeu RS, Lunga PK, Kuiate JR (2013). In vitro and in vivo antidermatophytic activity of the dichloromethane-methanol (1:1 v/v) extract from the stem bark of *Polyscias fulva* Hiern (Araliaceae). BMC Complement Altern Med.

[CR65] Okonkwo CO, Ohaeri OC, Atangwho IJ (2019). Haematological changes in rats exposed to insecticidal oils from the leaves of *Cassia occidentalis* and *Euphorbia milii*. Heliyon.

[CR66] Oladeji OS, Adelowo FE, Oluyori AP, Bankole DT (2020). Ethnobotanical description and biological activities of *Senna alata*. Evid Based Complement Alternat Med.

[CR67] Onlom C, Khanthawong S, Waranuch N, Ingkaninan K (2014). In vitro anti-Malassezia activity and potential use in anti-dandruff formulation of *Asparagus racemosus*. Int J Cosmet Sci.

[CR68] Onlom C, Nuengchamnong N, Phrompittayarat W, Putalun W, Waranuch N, Ingkaninan K (2017). Quantification of Saponins in *Asparagus racemosus* by HPLC-Q-TOF-MS/MS. Nat Prod Commun.

[CR69] Qiangqiang Z, Jiajun W, Li L (1998). Storage of fungi using sterile distilled water or lyophilization: comparison after 12 years. Mycoses.

[CR70] Ramesh VM, Hilda A (1999). Incidence of Keratinophilic fungi in the soil of primary schools and public parks of Madras City, India. Mycopathologia.

[CR71] Rahmani AH (2015). *Cassia fistula* Linn: potential candidate in the health management. Pharmacognosy Res.

[CR72] Ren X, Zhang Q, Zhang W, Mao J, Li P (2020). Control of aflatoxigenic molds by antagonistic microorganisms: inhibitory behaviors, bioactive compounds, related mechanisms, and influencing factors. Toxins.

[CR73] Rodrigues ML, Nosanchuk JD (2020). Fungal diseases as neglected pathogens: a wake-up call to public health officials. PLoS Negl Trop Dis.

[CR74] Salanţă LC, Tofana M, Socaci S, Mudura E, Pop C, Pop A, Cuceu A, Nagy M (2014). The potential of medicinal plants in developing functional foods. Hop Med Plants XXI.

[CR75] Salhi N, Mohammed Saghir SA, Terzi V, Brahmi I, Ghedairi N, Bissati S (2017). Antifungal activity of aqueous extracts of some dominant algerian medicinal plants. Biomed Res Int.

[CR76] Scorzoni L, de Paula e Silva ACA, Marcos CM, Assato PA, de Melo WCMA, de Oliveira HC, Fusco-Almeida AM (2017). Antifungal therapy: new advances in the understanding and treatment of mycosis. Front Microbiol.

[CR77] Shaban S, Patel M, Ahmad A (2020). Improved efficacy of antifungal drugs in combination with monoterpene phenols against *Candida auris*. Sci Rep.

[CR78] Sharafutdinov IS, Ozhegov GD, Sabirova AE (2020). Increasing susceptibility of drug-resistant candida albicans to fluconazole and terbinafine by 2(5H)-furanone derivative. Molecules.

[CR79] Sharma P, Bodhankar SL, Thakurdesai PA (2012). Protective effect of aqueous extract of *Feronia elephantum* correa leaves on thioacetamide induced liver necrosis in diabetic rats. Asian Pac J Trop Biomed.

[CR80] Sharma U, Kumar N, Singh B (2012). Furostanol saponin and diphenylpentendiol from the roots of *Asparagus racemosus*. Nat Prod Commun.

[CR81] Sharma U, Saini R, Kumar N, Singh B (2009). Steroidal saponins from *Asparagus racemosus (roots)*. Chem Pharm Bull (tokyo).

[CR82] Simonetti G, Brasili E, Pasqua G (2020). Antifungal activity of phenolic and polyphenolic compounds from different matrices of Vitis vinifera L. against human pathogens. Molecules (basel, Switzerland).

[CR83] Sitarek P, Kowalczyk T, Wieczfinska J (2020) Plant extracts as a natural source of bioactive compounds and potential remedy for the treatment of certain skin diseases. Curr Pharm Des. https://pubmed.ncbi.nlm.nih.gov/32303169/10.2174/1381612826666200417160049 32303169

[CR84] Sitarek P, Merecz-Sadowska A, Kowalczyk T, Wieczfinska J, Zajdel R, Śliwiński T (2020). Potential synergistic action of bioactive compounds from plant extracts against skin infecting microorganisms. Int J Mol Sci.

[CR125] Sony P, Kalyani M, Jeyakumari D, Kannan I, Sukumar RG (2018). In vitro antifungal activity of Cassia fistulaextracts against fluconazole resistant strains of Candida species from HIV patients. J Mycol Med.

[CR85] Srivastava PL, Shukla A, Kalunke RM (2018). Comprehensive metabolic and transcriptomic profiling of various tissues provide insights for saponin biosynthesis in the medicinally important *Asparagus racemosus*. Sci Rep.

[CR86] Tabanca N, Bedir E, Ferreira D, Slade D, Wedge DE, Jacob MR, Khan SI, Kirimer N, Baser KH, Khan IA (2005). Chem biodivers. Bioactive Const Turk Pimpinella Species.

[CR87] Taghipour S, Abastabar M, Piri F, Aboualigalehdari E, Jabbari MR, Zarrinfar H, Nouripour-Sisakht S, Mohammadi R, Ahmadi B, Ansari S, Katiraee F, Niknejad F, Didehdar M, Nazeri M, Makimura K, Rezaei-Matehkolaei A (2021). Diversity of geophilic dermatophytes species in the soils of Iran; the significant preponderance of *Nannizzia fulva*. J Fungi (basel, Switzerland).

[CR88] Tiwari RD, Singh J (1979). Anthraquinone rhamnosides from Cassia javanica root bark. Phytochemistry.

[CR89] Trease GE, Evans WC (2002). Pharmacognosy.

[CR90] Trottier CA, Jhaveri VV, Zimarowski MJ, Blair BM, Alonso CD (2020). Beyond the superficial: disseminated *Trichophyton rubrum* infection in a kidney transplant recipient. Open Forum Infect Dis.

[CR150] Tuknayat A, Bhalla M, Kaur A, Garg S (2020). Familial dermatophytosis in India: a study of the possible contributing risk factors. J Clin Aesthetic Dermatol..

[CR91] Upadhyay A, Chattopadhyay A, Goyary D, Mazumder PM, Veer V (2014). Topical application of *Cleome viscosa* increases the expression of basic fibroblast growth factor and type III collagen in rat cutaneous wound. BioMed Res Int.

[CR92] van Rhijn N, Bromley M (2021). The consequences of our changing environment on life threatening and debilitating fungal diseases in humans. J Fungi (basel, Switzerland).

[CR93] Wall G, Lopez-Ribot JL (2020). Current antimycotics, new prospects, and future approaches to antifungal therapy. Antibiotics.

[CR94] Wheat CM, Bickley RJ, Hsueh YH, Cohen BA (2017). Current trends in the use of two combination antifungal/corticosteroid creams. J Pediatr.

[CR95] Wójcik A (2016). Sandpits as a reservoir of potentially pathogenic fungi for children. AAEM.

[CR96] Wuthi-udomlert M, Kupittayanant P, Gritsanapan W (2010). In vitro evaluation of antifungal activity of anthraquinone derivatives of Senna alata. J Health Res.

[CR130] Yang L, Ren S, Xu F, Ma Z, Liu X, Wang L (2019). Recent advances in the pharmacological activities of dioscin. Biomed Res Int..

[CR97] Zagórska-Dziok M, Bujak T, Ziemlewska A, Nizioł-Łukaszewska Z (2021). Positive effect of *Cannabis sativa* L. Herb extracts on skin cells and assessment of cannabinoid-based hydrogels properties. Molecules (basel, Switzerland).

[CR98] Zahidin NS, Saidin S, Zulkifli RM, Muhamad II, Ya'akob H, Nur H (2017). A review of *Acalypha indica* L. (Euphorbiaceae) as traditional medicinal plant and its therapeutic potential. J Ethnopharmacol.

